# The prevalance, epidemiology and risk factors for onychomycosis in hemodialysis patients

**DOI:** 10.1186/1471-2334-7-102

**Published:** 2007-08-30

**Authors:** Güven Kuvandik, Meryem Çetin, Gultekin Genctoy, Mehmet Horoz, Mehmet Duru, Cenk Akcali, Salim Satar, Ahmet A Kiykim, Hasan Kaya

**Affiliations:** 1 Mustafa Kemal University, Faculty of Medicine, Department of Emergency Medicine, Hatay, Turkey; 2 Mustafa Kemal University, Faculty of Medicine, Department of Microbyology and Clinical Microbyology, Hatay, Turkey; 3 Mersin University, Faculty of Medicine, Department of Internal Medicine, Division of Nephrology, Mersin, Turkey; 4 Mustafa Kemal University, Faculty of Medicine, Department of Dermatology, Hatay, Turkey; 5 Cukurova University, Faculty of Medicine, Department of Emergency, Adana, Turkey; 6 Mustafa Kemal University, Faculty of Medicine, Department of Internal Medicine, Hatay, Turkey

## Abstract

**Background:**

Onychomycosis has a high prevalance among immunocompromised patients such as diabetics and hemodialysis patients. In the present study, we aimed to investigate the prevalence of onychomycosis among hemodialysis patients with and without diabetes mellitus, and to find out the factors likely to be associated with the development of onychomycosis among hemodialysis patients.

**Methods:**

One hundred and nine hemodialysis patients were enrolled. Fifty-seven of hemodialysis patients had the diagnosis of diabetes mellitus. Nail scrapings were obtained from 76 patients who had dystrophic nail changes. Samples were examined with 20% potassium hydroxide solution and all of the samples were inoculated on Saboraud's dextrose agar, potateus dextrose agar and mycobiotic agar. Diagnosis of onychomycosis was based on the presence of both positive clinical signs and positive potassium hydroxide test.

**Results:**

Onychomycosis was diagnosed in 26.6% of hemodialysis patients. Diabetes mellitus was present in 68.9% of patients with onychomycosis. Toenail scraping cultures were reported to be positive in 19.7% of patients with dystrophic nail changes. Logistic regression analysis revealed that the presence of diabetes mellitus and the mean duration of hemodialysis were the significant predictors associated with the development of onychomycosis.

**Conclusion:**

The prevalence of dystrophic nail changes and onychomycosis is increased among hemodialysis patients. The dialysis duration and the presence of diabetes mellitus are the independent risk factors associated with the development of onychomycosis in uraemic patients.

## Background

In general population, the prevalence of onychomycosis (OM) ranged between 2 to 11.1% [1-4]. Risk factors associated with the development of OM include increasing age, immunosupression, the presence of diabetes mellitus, family history, peripheral vascular disease, and disorders related to the skin such as hyperhidrosis, psoriasis, onychogriposis and nail trauma [1,5-8].

Among diabetic patients, the reported prevalence of OM ranges between 1.2–26% [9-11]. In a multicentre study, the risk odds ratio for diabetic subjects to have toenail onychomycosis has been reported as 2.77 times compared with normal individuals [9]. Patients with chronic renal failure may exhibit various cutaneous abnormalities including pruritus, xerosis cutis, alterations in cutaneous pigmentation, actinic elastosis and Raynaud's syndrome [12]. Increased frequency of a spectrum of nail disorders such as half-and-half nails, absence of lunula and splinter hemorrhage are also reported to be increased in chronic renal failure patients [13-15]. The frequency of OM in hemodialysis (HD) patients has been shown to be higher than healthy controls with a prevalence of 6.2–52% [14,16]. In addition, onychomycosis has been reported to be the second most frequent nail disorder in dialysis patients [16].

Although increased prevalence of OM has been noted in both patients with diabetes mellitus (DM) and patients with chronic renal failure on hemodialysis, there is no data related to the prevalence of OM among hemodialysis patients with diabetes mellitus. Therefore, in the present study, we aimed to investigate the prevalence of OM among HD patients with and without DM, and to find out the factors likely to be associated with the development of toenail OM among HD patients.

## Methods

### Subjects

One hundred and nine (35 females and 74 males; mean age: 54.6 ± 16.7) HD patients were enrolled in the study. All HD subjects consisted of patients with end-stage renal disease (ESRD) (creatinine clearance ≤ 5 mL/min/1.73 m^2^BS), who underwent HD treatment thrice weekly for 4 hours/day with blood flow rates of 200–250 mL/min and dialysate flow rates of 480–500 mL/min using bicarbonate dialysate on hollow-fiber artificial kidneys. The mean dialysis duration was 2.8 ± 2.7 years. The etiology of end stage renal failure were diabetic nephropathy in 36 patients (33%) and non-diabetic nephropathy in 73 patients (67%). Among patients who had non-diabetic nephropathy, 21 patients have been diagnosed to have diabetes mellitus according to American Diabetes Association criteria revised in 2004 [17]. Eventually, a total of 57 study patients (52.3%) had the diagnosis of DM on the time of the study.

The rate of treatment with insulin and oral hypoglycemic agents among diabetic patients were 52.6% and 47.4%, respectively. The study protocol was carried out in accordance with the Helsinki Declaration as revised in 1989 and approved by the Mustafa Kemal University Medical Faculty institutional review committee. All patients were informed about the study protocol and the written consent was obtained from each one.

### Exclusion criteria

Exclusion criteria included the history of collagen vascular disease, active infection, malignancy, alcohol abuse, cirrhosis, human immune deficiency virus infection, and the usage of immunosuppressant.

### Blood sample collection

Blood samples were drawn before a mid-week dialysis session after an overnight fasting at 08:00 pm to determine fasting blood glucose and glycosylated hemoglobin (HbA1C) levels (Beckman-Coulter Synchron LX20 Clinical. System; Beckman-Coulter, Fullerton, CA, USA).

### Mycological evaluation

After a detailed examination of the fingers and toenails, nail scrapings were obtained from 76 patients who exhibited dystrophic nail changes. The mycological studies included direct microscopic examination and culture. The nails were cleaned using 70% ethanol before sampling. Samples were obtained two times for each patient on different days. Microscopic examination of the nail scrapings were performed with the use of 20% potassium hydroxide (KOH) solution. Samples were cultured on Sabouraud dextrose agar, potato dextrose agar and mycobiotic agar (Sigma Chemical Co., St. Louis, Mo., USA). Cultures were incubated at 26°C and examined twice a week for a total duration of 4 weeks. The identification of dermatophytes was based on their macroscopic examination of fungal colonies and microscopic examination with lactophenol cotton blue dye. Yeast species were identified using germ tube test, selective agar CHROMagar Candida and API system ID 32C (bioMerieux Marcy l'Etoile, France). Moulds were considered as pathogens in the presence of following findings;

-the absence of the other organisms growth at the same culture media,

-the presence of mould growth at 3 samples,

-the presence of filaments identified at direct examination,

-improvement with antifungal therapy.

The diagnosis of OM was based on the presence of both positive clinical signs (dystrophic nail changes) and positive KOH test (OM positive group). Patients with negative KOH test and patients who had no dystrophic nail changes were grouped together as OM negative patients.

### Statistical analyses

The SPSS for Windows 11.0 program (SPSS Inc, Chicago, Illinois) was used for statistical analysis. A p value < 0.05 was considered statistically significant. Qualitative variables were assessed by Chi-square test. The multivariate logistic regression analysis was used to determine the association of independent variables such as age, gender, duration of dialysis, diagnosis of DM, hemoglobin A1C level, fasting blood glucose level, the presence of hypertension, the type of antidiabetic and antihypertensive medication, and the presence of diabetic nephropathy with the development of OM.

## Results

Dystrophic nail changes was observed in 46 (81%) diabetic and 30 (57.7%) non-diabetic HD patients p < 0.05). OM was diagnosed in 29 (26.6%; OM positive group) out of 109 HD patients according to the presence of both positive clinical signs (dystrophic nail changes) and positive KOH test. OM negative group consisted of 80 patients. DM was present in 20 (68.9%) patients of OM positive group. Three out of 29 OM positive patients (10.3%) and 8 out of 80 OM negative patients (10%) had a positive family history of OM (p > 0.05). None of the patients had a history of nail trauma, tight and high heeled or traumatic shoes, and excessive sweating of foot during activity.

Toenail scraping cultures were reported to be positive in 15 (%19.7) out of 76 patients who exhibited dystrophic nail changes. In 13 culture positive patients, the isolated fungi were dermatophyte and non-dermatophyte in 13 (86.7%) and 2 (13.3%), respectively (Table 1).

**Table 1:** The frequency of isolated fungi toenail scraping cultures

**Isolated fungi**	**Number of cultures**	**Type of fungi**	**Positive cultures (%)**	**KOH (+) patients (%)**	**Patients with DNC (%)**
*T. rubrum*	10 (9.2%)	D	51.7	34.5	13.2
*T. mentagrophytes*	2 (1.8%)	D	13.3	6.9	2.6
*Epidermophyton floccosum*	1 (0.9%)	D	6.6	3.4	1.3
*Aspergillus niger*	1 (0.9%)	ND	6.6	3.4	1.3
*Pichia etchellsii/carsonii*	1 (0.9%)	ND	6.6	3.4	1.3
Contamination	3 (2.8%)	-	-	10.3	3.9
Negative	58 (53.2%)	-	-	-	76.3

T, Trichophyton; D, Dermatophyte; ND, Non-dermatophyte; KOH, potassium hydroxide; DNC, Dystrophic nail changes.

**Figure 1:**
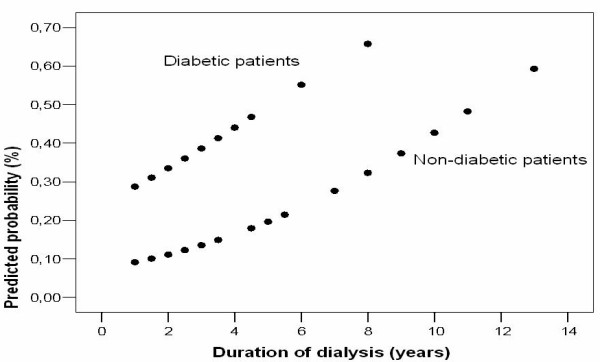
Predicted probability of onychomycosis according to duration of dialysis in the diabetic and nondiabetic patients.

Logistic regression analysis revealed that the presence of DM and the mean duration of hemodialysis were the significant predictors associated with the development of OM (Table 2). The predicted probabilities of the development of OM according to the duration of dialysis were plotted in diabetic and non-diabetic subjects (Figure 1). The other independent variables such as age, gender, hemoglobin A1C level, fasting blood glucose level, the presence of hypertension, the type of antidiabetic and antihypertensive medication, and the presence of diabetic nephropathy were not correlated with the development of OM (all p > 0.05).

**Table 2:** Logistic regression analysis determining the significant predictors of the development of onychomycosis

**Variable**	**Significance (p)**	**Relative risk**	**95% CI**
**Diabetes mellitus**	0.009	4.0232	1.417–11.424
**Duration of dialysis (years)**	0.008	1.2499	1.059–1.475

CI, confidence interval.

Logit (OM) = -2.52 + 1.39 (Diabetes) + 0.223 (Duration of dialysis)

## Discussion

In the present study, in concordance with the previous reports [13,14,18,19], we showed that the prevalence of abnormal appearing toenails and OM were increased among HD patients. In addition, HD patients with DM had higher dystrophic nail changes and OM prevalence compared to HD patients without DM. However, as the prevalence of dystrophic nail changes and OM prevalence among HD patients with DM have not been previously reported, we could not compare our findings with the literature findings.

In HD patients, increased susceptibility to nail disorders and OM may be due to both impaired immunity and histological changes of the skin caused by uremia. Histological alterations of the skin in uremic patients include severe microangiopathy and pericollogenous deposition of amyloid, identified as beta 2-microglobulin amyloidosis [20,21]. In venules and arterioles, endothelial cell activation and/or necrosis, basement membrane zone thickening and reduplication of basal lamina was also noted [22].

Previous studies have demonstrated that the prevalance of nail disease increased with HD duration [16,18]. Furthermore, Saray et al. demonstrated a possible relationship between HD duration and prevalance of OM among HD patients [19]. In concordance with those reports, in the present study, we observed that dialysis duration was an independent risk factor associated with the development of OM.

In comparison with the general population, patients with DM are predisposed to mycotic infections such as mucormycosis, and Candida infection of the mucous membranes, nails and skin folds [23]. Besides the high blood glucose levels and impaired immunity, diabetes related medical conditions may also provide contribution to the development of OM in diabetic patients [24]. For example, peripheral vascular disease results in impaired wound healing and increased risk of infection, and peripheral neuropathy results in decreased ability to detect the presence and the progression of infection [25].

Although conflicting reports [26,27] are available, patients with DM have been reported to prone to dermatophyte infections [28,29]. In addition, in two recent large scale studies [9,11], the prevalence of OM has been shown to be significantly higher in diabetic patients than normal individuals and increasing age, male gender, family history of onychomycosis, concurrent intake of immunosuppressive agents and peripheral vascular disease have been shown as independent risk factors for the development of OM. In contrast, in the present study, we did not find any correlation of increasing age, male gender, and family history of onychomycosis with the development of OM. This difference may be, in part, due to distinct characteristics of our patients (uremia plus DM). As we excluded patients receiving immunosuppressive agents and we did not investigate the presence of peripheral vascular disease in our patients, we could not make a comparison regarding to the influence of those parameters on the development of OM.

In the present study, *Trichophyton rubrum *was the most frequently isolated organism from the cultures and it was followed by *T. mentagrophytes*. These findings were in concordance with the previous studies conducted in diabetics without renal disease and normal population [9,10]. Interestingly, in the present study, *Pichia etchellsii/carsonii*, a non-pathogen yeast fungi, was isolated in one HD patients with DM. Although it may be an incidental colonization (not an infection) of toenail, it should be kept in mind that this non-pathogen yeast may become a potential pathogen in the presence of immunosupression caused by both DM and uremia.

## Conclusion

The prevalence of dystrophic nail changes and OM is increased among HD patients. The dialysis duration and the presence of DM are the independent risk factors associated with the development OM in uremia patients. As fungal infections have a potential progressive nature and dangerous serious outcome including erysipelas and in long term even amputation, especially HD patients with diabetes mellitus should be periodically examined for OM. In addition, education of the diabetic hemodialysis patients about the importance of foot and nail care should be an essential components in the management of those patients.

## Abbreviations

OM, onychomycosis; HD, hemodialysis; DM, diabetes mellitus; ESRD, end-stage renal disease; HbA1C, glycosylated hemoglobin; KOH, potassium hydroxide.

## Competing interests

The author(s) declare that they have no competing interests.

## Authors' contributions

GK, MC, GG, MH: Conception and design; MD, CA, SS, HK: Analysis and interpretation of the data; GG, GK, MD, HK: Drafting of the article; MC, MH, CA, SS, HK, AAK: Critical revision of the article for important intellectual content; GG, GK, MD, HK: provision of study materials or patients; GG, GK: Statistical expertise; GK, MC, MD, CA, SS: Collection and assembly of data. All authors read and approved the final manuscript.

## Pre-publication history

The pre-publication history for this paper can be accessed here:


